# Calpainopathy (Leyden-Mobius Limb-Girdle Muscular Dystrophy Type 2A Phenotype) and Dysferlinopathy (Miyoshi Distal Myopathy Limb-Girdle Muscular Dystrophy Type 2B Phenotype) of Preadolescent Onset: Case Reports of Two Male Filipinos

**DOI:** 10.7759/cureus.21353

**Published:** 2022-01-18

**Authors:** Joanna May S Quilacio, Raymond L Rosales, Encarnita R Ampil

**Affiliations:** 1 Department of Neurosciences and Behavioral Medicine, University of Santo Tomas Hospital, Manila, PHL; 2 Department of Neurology and Psychiatry, University of Santo Tomas Hospital, Manila, PHL

**Keywords:** dysf, capn3, miyoshi myopathy, leyden-mobius, dysferlinopathy, calpainopathy, lgmd2b, lgmd2a

## Abstract

Limb-girdle muscle dystrophy (LGMD) is the fourth most common genetic cause of muscle weakness, with LGMD type 2A (LGMD2A) being one of the most common adult-onset muscular dystrophies presenting with limb-girdle weakness, while LGMD type 2B (LGMD2B) being the most common distal myopathy.

This study includes two cases. The first case is a 13-year-old male, with no family history of similar symptoms, who presented with lower extremity weakness at the age of nine, starting with proximal weakness of the lower extremities, progressively involving the upper extremities. He had scapular winging and contracture of both Achilles tendons. The second case involves a 19-year-old male, with a distant family history of weakness, who presented with lower extremity weakness at the age of 10. He had distal myopathy, mainly as foot drop and atrophic gastrocnemii.

In both cases, cardiac, intelligence, and bulbar function are spared. Electroneuromyography (ENMG) for both revealed myopathic process. Genetic testing results revealed calpain 3 (CAPN3) and dysferlin (DYSF) abnormality, confirming the diagnosis of LGMD2A and LGMD2B, respectively.

This will be the first of its kind adequately documenting two of the most common LGMD subtype in our locale. Clinical phenomenology and preferential muscle involvement lead one to the gold standard genetic testing in heritable myopathies, which was well established in this report.

## Introduction

Limb-girdle muscular dystrophy (LGMD), first described around the late 18th century, is now a spectrum of 30 subtypes that have diverse manifestations due to different genetic involvement and genotype-phenotype variation [1-3]. Regardless, there are some distinguishing clinical characteristics of many LGMD disorders that may serve as guides for diagnosis [3].

LGMD is the fourth most common genetic cause of muscle weakness with an estimated minimum prevalence of one in 20,000 [4]. LGMD type 2A (LGMD2A)/R1 (calpain 3 mutation) is one of the most common adult-onset muscular dystrophies presenting with limb-girdle weakness [3,5]. While Miyoshi distal myopathy (LGMD type 2B (LGMD2B)/R2, dysferlin mutation) is the most common distal myopathy. LGMD2 disease subtypes are both autosomal recessive in inheritance. Nevertheless, they are the most common LGMD disorders [3,6].

Similar incidence was reported in China and Japan, with LGMD2A being the most common subtype, followed by LGMD2B [7,8]. Locally, there is only one report that was written and published. It involves a 43-year-old Chinese-Filipino male with distal lower extremity weakness of adolescent onset, with a sibling of similar complaints [9].

We, therefore, present two genetically confirmed cases of the two most common LGMD subtypes, LGMD2A and LGMD2B, and furthermore discuss the clinical phenomenology for both cases, as this governs the diagnosis and subsequent management for such cases.

## Case presentation

Case 1

A previously healthy Filipino male had his first symptom at 10 years old, which manifested as tiptoeing. He has no related comorbidities, and developmental milestones were at par with age. No other complaints were noted at this moment, and the patient can do activities of daily living. The weakness of lower extremities progressed, needing hand support for knee extension while going up the stairs. There was also difficulty standing from a seated position. Nevertheless, he can walk without assistance and continue his daily activities.

At the age of 13, the patient had difficulty carrying heavy objects and was easily fatigued. There was already a decline in his ability to perform daily activities such as difficulty in grooming. These symptoms prompted consultation with a neurologist.

Upon examination, he had atrophic proximal muscle groups, hypotonia, and hyporeflexia in the upper and lower extremities. Bilateral scapular winging was noted, and heel cords were tight. He exhibits generalized weakness; however, weakness was more prominent in the proximal muscle groups of the extremities. On standing from a seated position, Gower’s sign was demonstrated. There were long tract signs. The mental status and bulbar, sensory, autonomic, and other systemic functions were spared.

**Figure 1:**
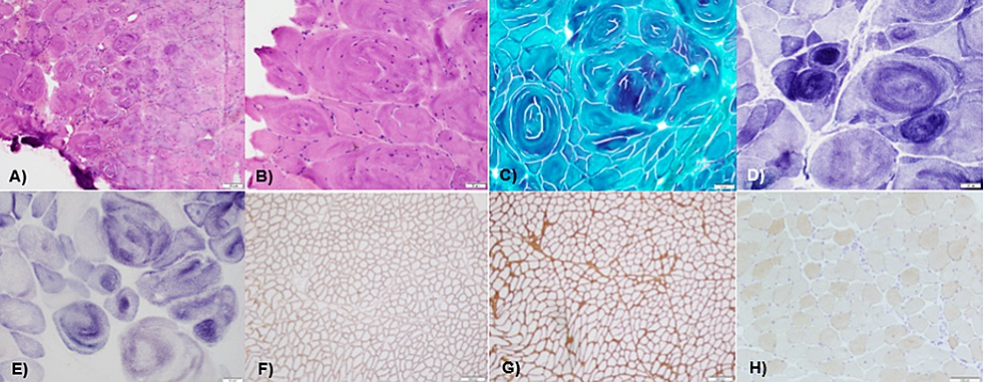
Left biceps brachium needle biopsy. A&B) H&E section with marked variation in fiber size with large hypertrophic fibers containing bizarre whorling of the sarcomere, internal nuclei, and incomplete fiber splitting and few necrotic and regenerating fibers with a mild increase in endomysial and perimysial connective tissue elements. C) Gomori trichrome stain negative for ragged red fibers or rimmed vacuoles. D) Nicotinamide adenine dinucleotide (NADH) and E) succinate dehydrogenase (SDH) stain with targetoid and whorled fibers. Immunohistochemical staining showing CAPN F) normal distribution of dystrophin and G) collagen IV and H) absent calpain.

The total creatine kinase (CK) of this patient was >3200 U/L.­­ Other examination results were also normal, including thyroid function examination and 2D echocardiogram. While electroneuromyography (ENMG) showed myopathic process. Left biceps brachium needle muscle biopsy was performed; the findings, however, were nonspecific but consistent with dystrophic process (Figure 1). Genetic analysis was done, which revealed two heterozygous pathogenic variants in the calpain 3 (CAPN3) gene, including nonsense mutation at exon 21, and a missense mutation from arginine to cysteine at exon 1.

Case 2

A 19-year-old Filipino male presented with progressive weakness of bilateral lower extremities. Developmental milestones were at par with age, and he had no comorbidities. The symptoms started at the age of 11, with progressive weakness of both lower extremities characterized as difficulty in running and dragging of his feet. This was despite muscle manipulation, consultation with a physiatrist, and physical therapy.

The patient was then referred to a pediatric neurologist at the age of 16 due to the persistence and progression of symptoms. CK and ENMG were requested. He was subsequently referred to a geneticist. Gene testing for dystrophin gene, however, was negative. At the age of 18, the patient noted a decrease of muscle mass in both gastrocnemius muscles, progressing proximally to both thighs.

**Figure 2:**
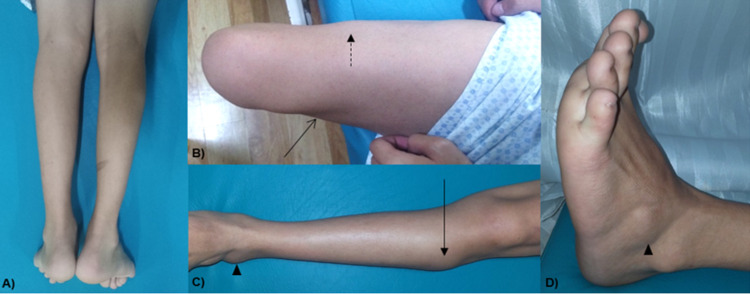
Examination of the lower extremities of the patient demonstrating A) atrophy of the posterior compartment of the legs, B) beginning diamond quadriceps (broken line) or atrophy of the vastus lateralis, sparing of the vastus medialis (open arrow), C) sparing of the lateral compartment of the legs (solid arrow), C&D) sparing of the extensor digitorum brevis (arrowheads).

On examination, both lower extremities had decreased muscle tone, and there was hyporeflexia (Figure 2). Marked atrophy was noted in both gastrocnemius muscle, with notable sparing of the lateral compartment of the legs and extensor digitorum brevis (Figure 2). Proximally, there was beginning atrophy of the vastus lateralis, with sparing of the vastus medialis (Figure 2). Both upper extremities were not affected. The weakness in both lower extremities was primarily distal, with prominent weakness in plantar flexion. Foot drop was seen bilaterally. Hip flexion and knee flexion also demonstrated weakness. There were long tract signs. The mental status and bulbar, sensory, autonomic, and other systemic functions were spared.

There was a distant family history of language and motor delay and a similar presentation of weakness among distant relatives.

Total CK was significantly elevated at >3200 U/L. Both ALT and AST were also elevated. Other examination results were also normal, including thyroid function examination and 2D echocardiogram, while ENMG showed myopathic process.

While muscle biopsy was contemplated, the patient opted to forego the procedure to prioritize resources on genetic analysis, with the leading and pathognomonic clinical phenomenology in mind. Genetic analysis showed a homozygous pathogenic variant at dysferlin (DYSF) gene creating nonsense mutation at exon 42.

## Discussion

LGMD2A, as seen in case 1, presents with proximal weakness and atrophy, with sparing of facial and neck muscles. The most common clinical presentation of this condition includes scapular winging and scoliosis. Achilles tendon contracture and other joint involvement are also common manifestations that can be prominently seen early at the start of the disease process. Rarely, patients may develop abdominal wall hernia as the consequence of the weakness of the external oblique muscles [3,10]. The patient described earlier manifested typical tiptoeing, ankle contractures, and scapular winging.

Three phenotypes have been identified based on clinical manifestations, particularly the distribution of muscle weakness and the age of onset [10]: 1) Leyden-Mobius (pelvifemoral) LGMD phenotype, which is the most common phenotype and in which weakness is prominently evident in the pelvic girdle and subsequently in the shoulder girdle, with onset often before the age of 12 years or after the age of 30 years; 2) Erb (scapulohumeral) LGMD phenotype, in which weakness is first evident, predominantly in the shoulder girdle and later in the pelvic girdle, with a milder variant having early age of onset; and 3) hypercreatine kinasemia (asymptomatic individual), with high serum creatine kinase (CK).

This patient may be classified under the Leyden-Mobius phenotype of LGMD2A, as it coincides with the onset in the first decade of life and initial and predominant lower extremity involvement.

LGMD2A is secondary to mutations in the CAPN3 (15q15.1-q21.1) gene, located in the long arm of chromosome 15 at position 15.1-21.1. The c.550delA mutation was initially thought to be a mutational hot spot, as it is the most common allele in the Caucasian population and was also found to account for 75% of mutant alleles in European countries [11]. Reports showed about 300-490 mutations in the CAPN3 gene, which are mostly (60%-70%) missense mutations [11].

The genetic study of the patient in case 1 revealed two pathologies in both copies of strands of CAPN3. One pathology is a missense mutation of amino acids arginine to cysteine at position 49 (NM_000070.2(CAPN3):c.145C>T p.Arg49Cys) and has only three previous registrations of the same mutation, one of which is pathogenic with the diagnosis of LGMD2A, and two registries are likely pathogenic [12]. The other copy is a nonsense mutation, at position 748 (NM_000070.2(CAPN3):c,2242C>T p.Arg748Ter), and has two pathogenic registration of the same mutation [12].

LGMD2B, on the other hand, also known as dysferlinopathy, has two clinical phenotypes based on clinical manifestations, particularly the distribution of muscle weakness [2,3,10]: 1) LGMD2B (proximal onset) phenotype, with proximal weakness with progressive distal involvement, and 2) Miyoshi distal myopathy (distal onset) phenotype, which is previously of a different category due to the involvement of distal extremities (in contrast to LGMD diseases, which are proximal). However, mutation of the DYSF gene was also the underlying pathology for this disease and hence was included in LGMD2B.

This initially involves the gastrocnemius and is later manifested by the inability to do plantar flexion and the presence of calf wasting. Progressively, the hamstrings and hip flexors are involved, and it is described by difficulty in climbing the stairs or rising from the ground.

In both phenotypes, the upper extremities will be involved at the latter stage. Muscles may show selective atrophy (e.g., selective atrophy of the vastus lateralis and rectus femoris, which accounts for the diamond sign on the lateral surface of the thigh, and the biarticular muscles). Other rare manifestations include distal myopathy with anterior tibial onset (DMAT) and intense calf pains and tenderness, followed by muscle wasting, asymptomatic hypercreatine kinasemia, and camptocormia. Patients with this condition have symptom onset in the second decade of life [13].

The patient in case 2 presented with calf pain, weakness, and atrophy of the lower extremities, with a predominance of atrophy of both gastrocnemii, with some level of selective wasting (Figure 2). The patient also had difficulty in plantar flexion with predominantly distal weakness. These findings are typical of Miyoshi muscular distal myopathy.

LGMD2B is a condition that results from the deficiency of the sarcolemmal protein dysferlin from mutation of the DYSF (2p13.2) gene, which is located at the short arm of chromosome 2 at position 13.2 [3,7,13]. Most mutations in this location result from nonsense mutation or abnormal truncation of dysferlin protein [12]. This is demonstrated by the genetic testing done in case 2, wherein the patient had a nonsense mutation at the region of the DYSF gene, at position 1517. (NM_001130987.1(DYSF):c.4551G>A p.(Trp1517*)). This genetic description is one of three variants, with its preferred identification as NM_003494.3(DYSF):c.4434G>A (p.Trp1478Ter). All variants have resultant truncation of dysferlin protein. There are four genetic registries of the same multivariants, one of which is likely pathogenic and the rest is pathogenic. There is no known mutational hot spot in this gene, but there have been founder mutations recorded across various populations worldwide [13].

Both calpain and dysferlin are not expressed in the cardiac myocytes. Hence, cardiac involvement among patients with these mutated proteins is rare [3].

Muscle histology, immunohistochemistry, and immunoblotting may be done. The pathologic features of LGMD diseases mostly are nonspecific and may vary. Most of them may include the presence of dystrophy, myopathy, neuropathy, inflammation, evidence of chronicity and disease progression, and signs of activity. Other findings include rimmed vacuoles and ragged, red, ring, and moth-eaten fibers [8].

Currently, there is no treatment for LGMD diseases. Management is directed toward symptoms, surveillance of probable consequences, and most importantly improvement of quality of life [3].

## Conclusions

Both cases manifested their own specific clinical presentation and phenomenology, with some pathognomonic signs that may point to the diagnosis. Both cases were also subsequently confirmed by genetic testing. This highlights the importance of genetic testing as a confirmatory diagnosis for hereditary muscular diseases. With the preponderance of genetic development, the standard of diagnosis has shifted from biopsy and staining to genetic testing. Most importantly, routine staining of biopsied muscle yields nonspecific results. Other diagnostic modalities, such as immunostaining and immunoblotting, may be done. However, these serve as modalities that are second in line once genetic testing yields a negative result or if the latter is unavailable.

In the Philippines, there is a big dilemma regarding the diagnosis, management, and exposure to such cases. Nevertheless, thorough history-taking, assessment of family history, and knowledge about the proper diagnostic tests to be done will help in properly localizing and eventually diagnosing LGMD. Precise diagnosis is necessary for prognostication and more precise management.
